# Don't fear the (small) bite: A narrative review of the rationale and misconceptions surrounding closure of abdominal wall incisions

**DOI:** 10.3389/fsurg.2022.1002558

**Published:** 2022-11-23

**Authors:** Alexis Theodorou, Mark Banysch, Hakan Gök, Eva B. Deerenberg, Joerg C. Kalff, Martin W. von Websky

**Affiliations:** ^1^ Department of General, Visceral, Thoracic and Vascular Surgery, University Hospital Bonn, Bonn, Germany; ^2^ Department of Surgery, St. Bernhard Hospital Kamp-Lintfort, Kamp-Lintfort, Germany; ^3^ Hernia Istanbul, Hernia Istanbul®, Hernia Surgery Center, Istanbul, Turkey; ^4^ Department of Surgery, Franciscus Gasthuis en Vlietland, Rotterdam, The Netherlands

**Keywords:** laparotomy closure, small-bites, incisional hernia prevention, abdominal wall, wound complications

## Abstract

**Background:**

The most common complications related to the closure of abdominal wall incisions are surgical site infections, wound dehiscence and the development of an incisional hernia. Several factors relating to the surgical technique and the materials used have been identified and analysed over the years, as mirrored in the current recommendations of the European Hernia Society, but some misconceptions still remain that hinder wide implementation.

**Method:**

A literature search was performed in the PubMed and GoogleScholar databases on 15 July 2021 and additionally on 30 March 2022 to include recent updates. The goal was to describe the scientific background behind the optimal strategies for reducing incisional hernia risk after closure of abdominal wall incisions in a narrative style review

**Results:**

An aponeurosis alone, small bites/small steps continuous suture technique should be used, using a slowly resorbable USP 2/0 or alternatively USP 0 suture loaded in a small ½ circle needle. The fascial edges should be properly visualised and tension should be moderate.

**Conclusion:**

Despite the reproducibility, low risk and effectiveness in reducing wound complications following abdominal wall incisions, utilisation of the recommendation of the guidelines of the European Hernia Society remain relatively limited. More work is needed to clear misconceptions and disseminate the established knowledge and technique especially to younger surgeons

## Introduction

Every operation in the abdominal cavity begins with an incision in the abdominal wall to gain access and ends with the closure of the abdominal wall. The most common incision is the midline laparotomy which can be made rapidly and causes minimal damage to muscles, nerves and blood vessels (1). It provides access to the entire abdominal cavity and can be expanded according to intraoperative findings if needed. However, it is also the incision that is associated with the highest risk of development of an incisional hernia (2), which in turn is the most common long-term complication related with any closure of the abdominal wall. Aside from incisional hernia development, two other short term common complications related with the closure of the abdominal wall are surgical site infections (SSI) and fascial dehiscence (burst abdomen) (3). It has been shown that both entities result in a higher risk of incisional hernia development later on. SSI is an independent risk factor for both dehiscence and incisional hernia (4). Patients with an SSI are far more likely to have an incisional hernia (5) and are at higher risk for secondary infectious complications after incisional hernia repair, even in an otherwise “uncontaminated” wound (6). Respectively, an incisional hernia will develop in the majority of patients that presented a fascial dehiscence after initial closure (7).

The presence of an incisional hernia is associated with a higher readmission rate and subsequent operations and is thus associated with a significant financial burden on health systems. Gillion et al. estimated that a reduction of incisional hernia incidence after abdominal surgery in France by just 5% would result in a national cost saving of 4 million Euros annually (8). Furthermore, patients with incisional hernias experience a lower health-related quality of life (QoL) in physical activity parameters and an impaired body image (9).

The connection between healing of the laparotomy wound and development of the above complications has thus been well established over the years. A variety of factors influencing the healing process have been identified with the intention to develop strategies to reduce post laparotomy complications: These factors can be grouped in three main categories, (a) patient related, (b) surgeon or surgical technique related and (c) material related (1, 2, 7, 10–15) (Table 1).

**Table 1:** Factors influencing wound healing after abdominal wall closure (1, 2, 7, 10–15).

Patient related	Surgeon/technique related	Material related
**Obesity**	Incision site	Suture material
Smoking	Incision technique	
Diabetes	Suture technique	
Collagen deficiency	Wound contamination	
Immunosuppression	Postoperative Management	
Nutritional deficiency	Skin closure	
Clinical condition (sepsis, shock)	NPWT application	

Optimization of patient related risk factors requires, in theory, long pre-planning and screening before surgery rendering preoperative optimization extremely difficult for most cases. Scientific interest in “Prehabiliation” programs prior to major abdominal surgery increases of late and first studies show promising results in reducing overall and pulmonary morbidity (16) but definitive clinical effectiveness is currently very limited (17), especially regarding wound complications (18, 19). Hopefully, in the future, more research in this area will allow further reduction of postoperative wound-related morbidity.

Surgeon/technique related factors, are most directly modifiable and thankfully improvements of these factors are also most facile to implement. In general, closure techniques can be broken down into five separate factors (Table 2) or surgeon decisions. Each of them has been proven to have a positive influence in reducing wound morbidity and subsequently incisional hernia rates.

**Table 2:** Surgical technique related factors.

Technique related factors	Material related factors
1. Continuous vs. interrupted suture	i. Needle size
2. Suture length to wound length ratio	ii. Absorbable vs non Absorbable
3. Bite size	iii. Antibacterial coated/non coated
4. Mass closure vs. aponeurosis only	iiii. Monofilament vs. multifilament
5. Tension	

The European Hernia Society published guidelines for the closure of abdominal wall incisions for the first-time in 2015 (2) and updated them in collaboration with the American Hernia Society in 2022 (20). Considering all available evidence, the updated guidelines further suggest the use of running suturing with a slowly absorbable monofilament suture in a single layer, aponeurotic, small bite technique with a SL:WL ratio of 4:1 (2) for elective midline incisions. The guidelines -in its initial and updated versions- represent a big step towards harmonisation of abdominal closure technique, providing evidence-based and simple to follow steps for a problem with significant morbidity but are often overlooked by surgeons. In the years following the initial guidelines, a significant number of studies further defined the small bites technique and provided a clear, detailed and easy to follow procedure (21). In contrast to these findings and even though recent surveys showed that the small bites technique is reproducible, has no risks and provides low incidence of incisional hernia, a wide dissemination and “real world” implementation is still not optimal in the surgical community (22, 23). In the published literature, no specific explanations for this dogged acceptance of an empiriously validated technique can be found. From our personal experience, two main reasons may be hypothesized. First, prevalent misconceptions about the true nature of the “small bites technique” and the defined SL: WL ratio may lead many surgeons to the (often times) false conclusion that they already are performing it, and secondly- a mechanistic deep-rooted conception that “a thicker suture is more stable and will hold more”. The guidelines, in both initial and updated version, though they represent a comprehensive review and summary of all available clinical data to date, don't directly mention the “mechanistic” background of closure techniques that is in our opinion critical in addressing some of the fears surgeons have regarding the “small bites” closure.

In this narrative review of the current literature, best practice and past science, we intend to deconstruct those misconceptions and provide surgeons with a practical viewpoint and explanation for the rationale behind the “small bites” abdominal closure technique.

## Methods

A literature search was performed in the PubMed and GoogleScholar databases. The search was restricted to the English and German language. No restriction on year of publication was set. The following keywords were used [abdominal wall closure, wound complications, incisional hernia prevention, laparotomy closure, wound closure techniques, burst abdomen]. After duplicates were identified, the resulting articles were screened by title and abstract. The articles were critically evaluated regarding relevance to the aim of the review and quality. This was performed by the primary author. Additional suggestions were possible from the other authors. In case of disagreement the senior author was consulted. Original articles were preferred over other narrative review, though, selected editorials from key opinion leaders were included. A snowball strategy was then applied, where citations of the selected articles were screened for to identify publications of importance. Time of initial search was July 15, 2021. A complimentary search war performed in March 30, 2022 to include recent updates.

## Technique matters

### Continuous vs. Interrupted suture

In 2010 Diener et al. published the INLINE review (24) enrolling a total of 14 RCTs and 5 previously published systematic reviews where it was shown that a continuous suture technique (with a slowly absorbable suture) results in significantly lower chance of developing an incisional hernia. The strong evidence provided by Diener was later reflected in the recommendations of the EHS about closure of abdominal wall incisions. A continuous suture has since then been established as the gold standard. In a recent study by Bloemen et al., as many as 98% of the participating surgeons used running sutures (3). The assumption here is that a continuous suture results in a preferable distribution of tension along the wound edges. Optimal tension distribution results in a significantly higher accumulation of collagen protein in the wound which is incremental for healing and scar formation (13, 25–27). During scar formation, the mechanically weak collagen type III will be gradually replaced with collagen type I that possess higher tensile strength (13). A continuous suture (with a SL/WL ratio >4:1) resulted in (a) earlier appearance and (b) larger extent of the preferable collagen fraction which is essential for the mechanical strength of the healing laparotomy (13). The principle of tension distribution is a recurrent principle, evident throughout the technical decisions described in this review.

At the point of this review, no studies could be found regarding the use of one suture for the entire wound vs. two sutures beginning from the opposite ends that get connected in the middle. Use of one or the other, depends on personal preference. The advantage of using two sutures, lies in the better visualisation of the fascial edges where the wound is at its most narrow and is favoured by the authors.

Nevertheless, closure by interrupted sutures is still in use in surgical practice around the world. Publications from India about emergency laparotomy in the presence of peritonitis suggest that, at least in the described setting, an interrupted suture may result in less risk of burst abdomen than continuous large bites sutures (28) while the short follow up (2 weeks) gives no information about the risk of developing an incisional hernia. Several studies and publications from Europe and the US though, show that closure by continuous suture leads to improved outcomes in an emergency setting as well (29–32). It is evident that more high quality studies that investigate the influence of suture technique in reducing wound complications in an emergency setting are needed. In the author's experience and opinion, closure by a continuous suture should be performed, if possible. Still, in complex cases of delayed primary closure after open abdomen treatment after peritonitis, the fascial structure and wound situation sometimes does not allow a dedicated continuous aponeurosis suture. In these difficult cases, an interrupted double “figure of 8” suture with the same slow-absorbing monofilament suture material, prepared after good visualisation of the fascial edges and tied one by one after all sutures are placed, can provide a solution.

### Suture-length: wound-length ratio. Steps and bites

The suture-length: wound-length (SL:WL) ratio measures the length of suture used to close the wound in comparison to the wound length. It is an excellent marker of the size of the “steps and bites” used in the suture. Bite size refers to the distance between the edge of the wound and the needle's point of entry. Step size refers to the distance between two consecutive stitches. In their ground breaking publications, Leif Israelsson et al. and Cengiz et al demonstrated that using at least 4 times suture length to wound length increases tensile strength and leads to a significant reduction of incisional hernia development and wound infections (1, 33). To meet the recommended SL:WL ratio a surgeon can either increase bite size (long stitch) or use smaller bites at more frequent intervals (small steps). In similar SL:WL ratio scenarios, small bites show superior wound bursting strength (34) in contrary to what seems to be the common belief among surgeons. The initial experimental findings were later confirmed by clinical trials, most notably the STITCH trial. Published in 2015 in the Lancet, this prospective, multicenter, double-blinded, randomised controlled trial by Deerenberg et al showed that a small bites suture technique resulted in reduced development of incisional hernia in elective midline incisions when compared with large bites (21). Although no significant reduction in wound infection rate or burst abdomen was observed in the STITCH trial, other studies showed that implementation of the small bites technique was correlated with a significant reduction in SSI (23, 35). Most significantly, no negative effects for the small bites technique were observed in any of the studies. A common complaint of surgeons after a long and demanding open procedure is the extra time needed for small bites closure in comparison with closure “as usual”. But mean additional closure time in the STITCH trial was 4 min. The reasoning behind this technique lies in the ideal distribution of forces on the fascia that leads to optimum ratio of collagen type I to collagen type III, similar to the continuous vs. interrupted suture discussed above (13). The combination of small bites and small steps (required for optimum SL:WL ratio) is shown in studies and meta-analyses to be effective in reducing late wound complications as incisional hernia (32, 36). The resulting closure has higher wound strength. A main advantage of this is the better distribution of tension. Less tension per suture results in less tissue tearing that creates loose points, or creates less problems with tissue blood supply due to strangulation (34).

The technique is shown to be safe and easy to learn but often the surgeon's subjective perception that the technique is performed correctly is misconceived. In a recent study from Rodriguez et al. only 30% of surgeons (which all had stated that they already performed the small bites technique correctly), really did so when objectively measured in their first try (3, 23) The number rose to 95% in the third attempt, which shows the importance and facility of a short course hands on training of the small bites technique. SL:WL ratio is the most prominent and easily monitored quality criterion of abdominal closure and a higher SL:WL ratio (>4:1) is directly related with reduced complication rates (1). It is our opinion that it should be measured and recorded individually at least once (!) in an effort to increase awareness and become the standardised technique. Ideally, to achieve the desired SL:WL ratio the suture steps should be 4–5 mm and the bites 5–8 mm (36). In a recent study Gregory Conway et al. published findings that have shown how difficult it is for not well trained surgeons to estimate the distance between stitches. If a small steps procedure has been required, the 5-mm mark placement estimates ranged from 2.01. to 11.69 mm (37). These results emphasize the importance of intraoperative measurement.

### Mass closure vs. aponeurosis only

The “mass closure” technique includes the aponeurosis and adjacent soft tissues in the suture. Usually this is a result of the long stich technique where subcutaneous fat and practically parts of the rectus abdominis muscle are voluntarily or involuntarily “stitched” together. After surgery, the intra-abdominal pressure is elevated. This can be moderate due to the termination of muscular relaxation or high for example due to coughing. This excessive stress on the suture line may result in the suture cutting through, or compressing the weaker tissues such as fat and muscle, creating the “button hole” effect (36) (Picture 1). This may lead to loosening of the suture or soft tissue necrosis, increasing the risk of complications such as dehiscence, incisional hernia or SSI (38). In both lab settings (38, 39) and clinical trials (3, 21, 36), an “aponeurosis only” suture as opposed to “mass suture”, results in increased wound tensile strength and reduced complication rates. The aponeurosis is the most stable part of the abdominal wall (40). Fat tissue has no contribution in terms of stability and muscle tears easily, so it should be no surprise that involving them in the stitch and closure can have no benefit: As one prominent Francophile surgeon once put it: “muscle sutue est muscle foutue” (loosely translated as “muscle sutured is muscle damned”). Furthermore, as a mostly avascular tissue, the aponeurosis is less vulnerable to ischemic damage due to strangulation from the suture. The correct technique would require to visualise 1 cm of aponeurosis on each side of the wound to ensure that no fat tissue will be included in the stitch (36). Perforating the muscle should also be avoided as the suture will cut though or compress easily. Stark compression of either fat or muscle tissue can also lead to secondary tissue necrosis, further compromising the stability of the closure (38).

### Tension

Another common misconception (and infamous common cause of drama between the surgeon and the first assistant) is the subject of suture tension. Instinctively, we tend to associate higher tension with a more stable closure. This “instinctive truth”, however has been repeatedly proven to be wrong: The working group of J. Höer has proven that a high tension suture can result in a lower tensile strength in the healed wound. Tissue perfusion after low tension closure is significantly higher than after closure with high tension (27). Tissue strangulation, sutures cutting through, oedema and necrosis are consequences of extreme tension that lead to an increase in short- and long-term wound complications. Similar to the other key points mentioned in this article, high tension sutures have a negative influence in collagen synthesis that leads to persistent high levels of the unstable collagen Type III (13).

Exact measure of suture strength is practically not possible during surgery, and the required tension may vary with structure and condition of the fascia. The wound edges should be adapted but not compressed (1). A good rule of thumb is that the stitches should be visible and not sink into the tissue. Unfortunately, this acquisition of tissue sensibility and the judgement of perfect tension comes to the surgeon only by experience which means trial and error and thus morbidity for the patient- unless we are able to install learning setting which enable young surgeons to understand the basics and practically improve their abdominal closure repertoire.

## Material matters

### (Needle) size matters

The material we use to close the abdominal incision may be of equal importance with how we close it. Choice of needle, suture size, absorbable or non-absorbable and coated or non-coated sutures can have a decisive effect on closure success but also a somewhat underrated effect on the technique being employed. What is becoming very clear is, that in order to combine all above mentioned steps, the needle size and suture strength is of great relevance. Respecting the recommended step and bite sizes with a “big” needle would be extremely difficult if not impossible, as the needle size presets if not dictates the stitching technique. A ½ Circle 48 mm needle regardless of surgeon skill, is not compatible with a small bites closure. To adequately perform the small steps and bites needed, a ½ circle tapered needle of maximal 31 mm is recommended.

Usually though, smaller needles tend to come attached to thinner sutures. At first glance, this goes against surgical instinct! Throughout surgery (and everyday life), high tension repairs are made with thick sutures. A USP 2/0 suture as the one used in the STITCH trial and recommended for a small bites closure has a lower breaking point compared to e.g., the USP 1 double loop. This leads many surgeons to disregard the clinical evidence about the safety and efficacy of the small bites technique (21, 41), in fear of suture failure and the embarrassing development of a burst abdomen- and to chose the thicker suture. Suture line strength though, as demonstrated by Jenkin's “spring coil theory”, relies on the spring coil effect the continuous suture creates, distributing the tension created from the pressure of the abdominal cavity in the entire length of the suture (42). A SL:WL ratio of at least 4:1 is thus vital on creating sufficient suture line tensile strength with a USP 2/0 suture. A possible solution to overcome such considerations would be the use a USP 0 suture mounted on a smaller 24 mm needle (like the CT-2 needle).

### Absorbable vs. non absorbable suture material

The European Hernia Society in the latest guidelines about closure of abdominal wall incisions recommends the use of a long-term absorbable suture (2). Healing of the aponeurosis is very slow compared with other tissue (40) and even a year after the incision the abdominal fascia retains only about 70% of its original strength (43). A rapid absorbable suture, Polyglactin (Vicryl) retains 75% of the tensile strength at 2 weeks and only 50% at 3 weeks. At this time, in a normal healing scenario the aponeurosis has reached only about 20% of its original strength and less than 10% in delayed healing scenarios (40, 42, 43). Therefore, after abdominal wall closure it is advisable to use a suture that provides extended wound support. Polydioxanone (PDS) retains 60% of the original tensile strength at 6 weeks. At this time the aponeurosis has reached more than 50% of original strength even in the delayed healing scenario. In a meta-analysis of 8 RCT encompassing 4,261 patients, Sajid et al. showed that PDS, Prolene and Nylon all are equally effective for abdominal fascial closure regarding risk of incisional hernia, wound dehiscence, peri-operative complications, suture sinus formation, and surgical site infection (44). At the same time absorbable sutures are known to be more comfortable for the patient and appear less likely to cause post-operative pain than non-absorbable sutures (43, 45). Therefore, with no additional gain and more possible side effects, non-absorbable sutures are not recommended for the closure of abdominal wall incisions in accordance with EHS guidelines.

### Infection control

Any implant, including a suture, increases the risk of bacterial colonisation and biofilm formation and may contribute to an infection. As discussed in the beginning of this article, presence of an SSI leads to a significant risk of development of both a burst abdomen and incisional hernia. Surgical site infections represent 14% of all nosocomial infections and 5% of all surgical complications (46). SSI after midline laparotomy reach up to 16%, prolonging hospital stay, increasing mortality and increasing health care costs. To effectively reduce the risk of SSI, the World Health Organisation issued a set of global guidelines for the prevention of surgical site infections in 2017 (47). The guidelines, recommend a number of actions as part of a bundle covering a variety of issues, as maintaining normothermia, nutritional support etc. Regarding the closure technique, the use of antimicrobial sutures is introduced as a possible solution. Sutures coated with Triclosan; a broad-spectrum antiseptic agent that reduces the risk of biofilm formation, have been shown in meta-analyses to reduce SSIs up to 28% (46, 48–51). These meta-analyses included different kinds of incisions, tissues and sutures and data regarding the risk of incisional hernia after midline fascial closure were less conclusive in establishing a clear benefit. Still, a clear reduction of SSI and a tendency towards lower risk of incisional hernia was observed in studies examining closure of abdominal incisions (52). Recognising the multiple benefits in SSI reduction both in terms of clinical effectiveness and cost effectiveness, the National Institute for Health and Care Excellence (NICE) issued at 28th of June 2021 a Medical Technologies Guidance (MTG) recommending the use of such coated sutures as part of a bundle of care for preventing surgical site infection in the NHS, for wound closure after a surgical procedure when absorbable sutures are an appropriate option (53).

## Current recommendations

Compiling all elements discussed above and based on the current data, this is our strategy in closure of abdominal wall incisions, in order to minimise wound complications (Pictures 2, 3).

1. A small bites/small steps continuous suture technique should be used. Bite size should be between 5 and 8 mm and step size no more than 5 mm. Suture length to wound length ratio should be measured at the end and be at least 4:1 or bigger.
2. The fascial edge should be properly visualised. Around 0.5–1 cm of aponeurosis on each side of the wound (incl. the cranial and caudal edges) can be freed to allow an aponeurosis only suture and muscle and fatty tissues should be pushed of the fascia with the needle when making a stitch.
3. We recommend using a slowly absorbable USP 2/0 or alternatively USP 0 suture loaded in a small ½ circle needle. If possible, especially in high-risk patients, a Triclosan coated suture could be used.
4. Moderate tension with the suture line still visible, not cutting in to the tissue.
5. Marking the skin prior to the incision in 1 cm steps in thin patients could provide a visual assistance for a small bites closure (a stitch at the marking and one between).

## Conclusion

In the past 20 years, significant progress has been made in our understanding of the factors that influence healing of elective abdominal wall incisions after closure. In the classic, elective laparotomy closure situation, the evolvement of the small bites technique and the move towards an aponeurosis only suture with a (slowly) absorbable, potentially antibacterial coated suture represents a major paradigm shift that has only few similar in surgery. Utilisation of this however still remains relatively limited. Clearly, more work is needed to disseminate the established knowledge and technique especially to younger surgeons. Dossa et al., identified five strategies for improving uptake of the small bites technique, including facilitated hands-on workshops and standardisation of closing processes (54).

Nevertheless, our understanding of the pathophysiology in high-risk situations like infected wounds or emergency laparotomies is still very limited. In most of the studies cited in this review, emergency laparotomies were excluded. At the same time, these patients are the ones that may profit the most from strategies to reduce wound complications which are very common in these cases. Potentially, while not the subject of this review, the use of barbed sutures (50) as well prophylactic mesh implantation have shown promising first results in such settings (55) with the updated guidelines of the European Hernia Society suggesting the latter in specific high risk situations. Further research, both primary and clinical, is needed to prevent post laparotomy wound complications in both elective and emergency settings such as emergency laparotomy and open abdomen treatment.

## Figures and Tables

**PICTURE 1:**
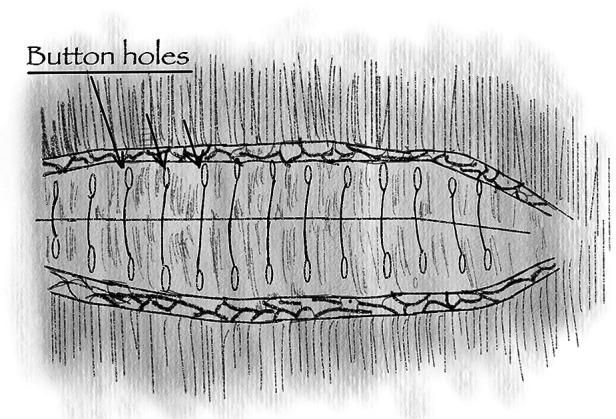
“Button hole” effect of sutures cutting through soft tissue caused through high tension.

**PICTURE 2:**
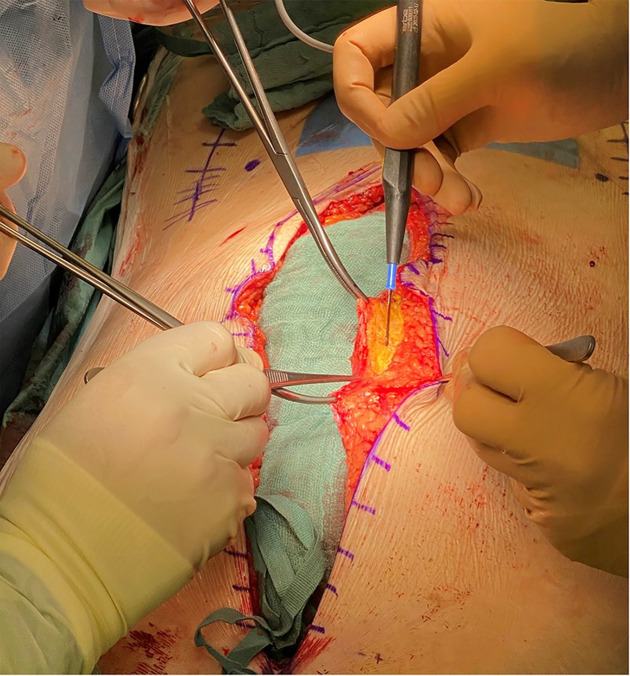
Preparing the fascial edges.

**PICTURE 3:**
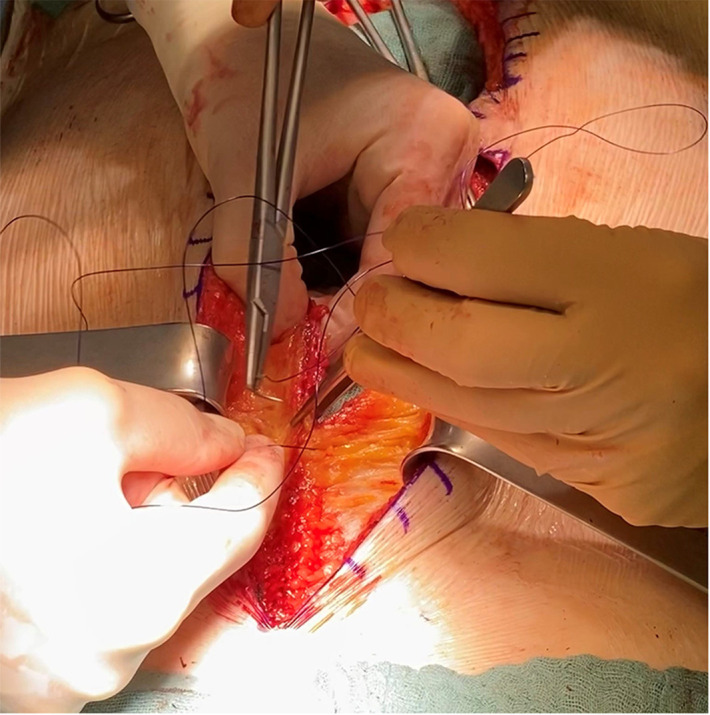
Using a USP 0 suture with a 26mm 1/2 circle (CT-2) needle in 5-8mm bites and 5mm steps to achieve a 4:1 SL:WL ratio. Tension on the sutures is kept moderate.

**PICTURE 4:**
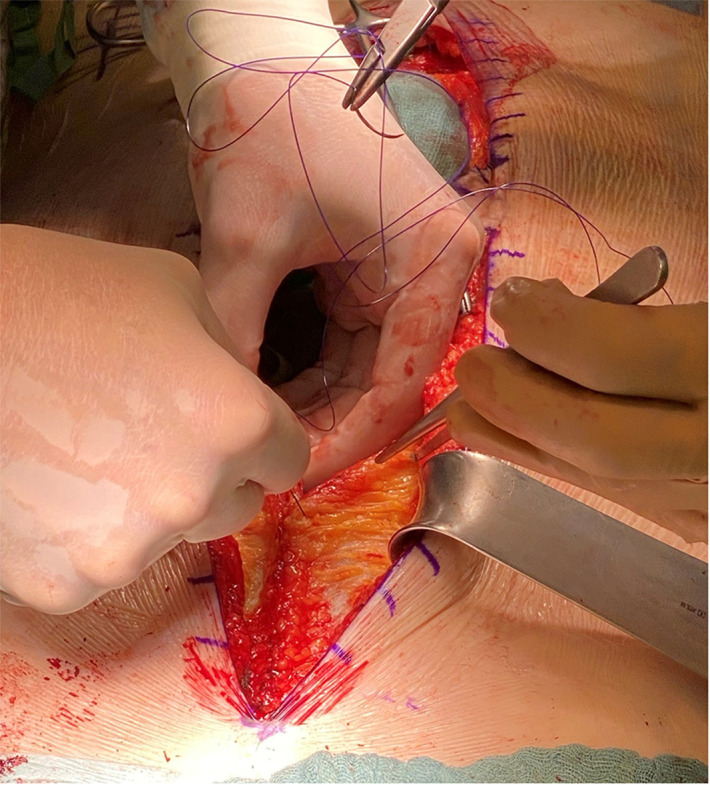
Using a USP 0 suture with a 26mm 1/2 circle (CT-2) needle in 5-8mm bites and 5mm steps to achieve a 4:1 SL:WL ratio. Tension on the sutures is kept moderate.

## Appendix

Pictures 1–4 belong to the personal archive of the first author, A. Theodorou. Informed consent was acquired and can be provided if requested.
